# Blood Vessels and Vascular Niches in Bone Development and Physiological Remodeling

**DOI:** 10.3389/fcell.2020.602278

**Published:** 2020-11-27

**Authors:** Michelle Hendriks, Saravana K. Ramasamy

**Affiliations:** ^1^Institute of Clinical Sciences, Imperial College London, London, United Kingdom; ^2^MRC London Institute of Medical Sciences, Imperial College London, London, United Kingdom

**Keywords:** blood vessels, niche, development, physiology, remodeling, microenvironment

## Abstract

Recent advances in our understanding of blood vessels and vascular niches in bone convey their critical importance in regulating bone development and physiology. The contribution of blood vessels in bone functions and remodeling has recently gained enormous interest because of their therapeutic potential. The mammalian skeletal system performs multiple functions in the body to regulate growth, homeostasis and metabolism. Blood vessels provide support to various cell types in bone and maintain functional niches in the bone marrow microenvironment. Heterogeneity within blood vessels and niches indicate the importance of specialized vascular niches in regulating skeletal functions. In this review, we discuss physiology of bone vasculature and their specialized niches for hematopoietic stem cells and mesenchymal progenitor cells. We provide clinical and experimental information available on blood vessels during physiological bone remodeling.

## Introduction

Bones, the structural and mechanical components of our body, are also involved in whole-body metabolism, brain functions, mineral homeostasis, and blood cell generation (Clarke, 2008; Bahney et al., 2015; Ramasamy, 2017). They are highly vascularized, metabolically active tissues having an extensive network of blood vessels except in cartilaginous regions (Clarke, 2008; Marenzana and Arnett, 2013; Lafage-Proust et al., 2015). Measurements in multiple animal species including humans estimate the proportion of cardiac output directed toward the skeletal system to be in the range of 5–15% (Marenzana and Arnett, 2013; Tomlinson and Silva, 2013; Prisby, 2017; Ramasamy, 2017). Such an enormous supply of blood indicates high nutrient demand, associated cellular processes, and the importance of blood vessels in bone and body homeostasis. Blood vessels are not just transport conduits; endothelial cells (ECs), the building units of blood vessel tubules, interact closely with the tissue cells to regulate their physiology and functions (Ramasamy et al., 2015). Several pieces of evidence confirmed the importance of blood vessels in regulating bone physiological functions such as blood cell production (hematopoiesis) and bone formation (osteogenesis). In this review, we discuss current understandings of bone vasculature, its niches and their role in skeletal health and disease.

## Blood Vessels During Bone Development

The process of bone formation begins *in utero* and continues into adult life in order to repair fractures and remodel bones based on physiological demands. During early development, initiation of bone formation from cartilage anlagen is associated with blood vessel invasion around embryonic day 14.5 (E14.5) (Kronenberg, 2003; Maes et al., 2010). After this, bone formation (ossification) begins and continues postnatally. Most long bones undergo both primary ossification of the diaphysis, which accompanies growth and begins *in utero*, as well as secondary ossification of the epiphysis which is independent and begins later in adolescence (Ortega et al., 2004).

Two distinct mechanisms of ossification, endochondral and intramembranous (Berendsen and Olsen, 2015), mediate bone formation in mammals. Endochondral ossification occurs in long bones such as the femur and tibia (Ben Shoham et al., 2016), which involves the formation of an intermediate cartilage structure. Intramembranous ossification is mostly involved in the formation of flat bones such as the skull, although both types of ossification can be used at different stages of development in the same bone, such as for the occipital bone (Jin et al., 2016). During intramembranous ossification, mesenchymal cells differentiate directly into osteoblasts without intermediate cartilage formation (Thompson et al., 1989). During both mechanisms, vascular endothelial growth factor (VEGF) attracts ECs to vascularize the bone tissue; vascularization coincides with the appearance of bone and hematopoietic cells (Chim et al., 2013; Ramasamy et al., 2015). However, the vascular invasion has been suggested to differ between endochondral and intramembranous ossification. In endochondral ossification, hairpin loops form from the perichondral vascular network, with a capillary glomerulus forming at its end; this then elongates into the bone, and other capillaries extend backwards from the loop to fully vascularize the tissue (Skawina et al., 1994). In intramembranous ossification, small capillaries extend into the mesenchyme surrounding the future ossification center, followed by a cascade of ossification and further vascularization (Thompson et al., 1989). It is also shown that the vasculature influences morphogenesis of developing bone; bone ECs are coated with collagen I, which serves as a template for osteoblasts to lay down mineralized bone (Ben Shoham et al., 2016).

Angiogenesis, the process of new blood vessel formation from an existing vessel, is closely coupled with osteogenesis by specialized type-H capillaries. This capillary subtype, so named due to high expression of both EC markers Pecam1 and Endomucin (Emcn), are present in the metaphysis and endosteum regions of bones. Expansion of type-H vessels by activating Notch or stabilization of hypoxia-inducible factor (HIF) in ECs leads to increases in trabecular bone, bone mass, and osteoprogenitor numbers (Kusumbe et al., 2014; Lafage-Proust et al., 2015; Ramasamy et al., 2016). Age-related decline in bone mass could be recovered by reactivating type-H capillaries in aged bones where apparent type-H capillaries are not observed. Developmental promotion of type-H ECs is achieved mainly through VEGF and is modulated by fibroblast growth factors (FGFs), bone morphogenic proteins (BMPs), and transforming growth factor β (TGFβ) (Chim et al., 2013; Kusumbe et al., 2014; Lafage-Proust et al., 2015; Peng et al., 2020). VEGF and a myriad of other angiogenic factors are produced by chondrocytes, osteoblasts, and to a lesser extent osteoclasts and osteocytes (Eriksen, 2010; Chim et al., 2013; Ramasamy et al., 2016; Peng et al., 2020). Loss of main isoforms of mouse VEGF-A leads to impairment of both vascularization and ossification during development (Maes et al., 2002; Zelzer et al., 2002). There is evidence that other mesenchymal cells also promote and participate in angiogenesis and arteriogenesis (Al-Khaldi et al., 2003). At the same time, EC-secreted angiocrine factors such as BMPs, FGFs, endothelin-1, and VEGF are involved in regulating osteoclast and osteoblast differentiation and function (Brandi and Collin-Osdoby, 2006; Eriksen, 2010; Romeo et al., 2019; Sivan et al., 2019). Angiocrine functions of bone endothelium are also associated with blood flow-mediated control of bone formation (Ramasamy et al., 2016).

Angiogenic type-H vessels support a new osteoclast subtype called vessel-associated osteoclasts (VAOs) which are non-resorbing osteoclasts involved in regulating angiogenesis. Type-H ECs express receptor activator of nuclear factor kappa-B ligand (RANKL) to regulate VAO formation through RANK signaling during developmental endochondral bone formation (Romeo et al., 2019). However, typical bone-resorbing osteoclasts are not significantly altered by type-H ECs. Blood vessels are considered to bring osteoclast precursors to the bone surface; meanwhile, osteoblasts may originate from circulating or perivascular osteoprogenitors (Maes et al., 2010; Lafage-Proust et al., 2015). Secretion of platelet-derived growth factor BB (PDGF-BB) by pre-osteoclasts recruits both ECs and osteoblasts, inducing type-H vessel formation and stimulating bone growth during remodeling (Xie et al., 2014; Lafage-Proust et al., 2015; Peng et al., 2020). Recently, SLIT3 from osteoblast lineage cells has also been identified to display similar effects (Xu et al., 2018).

## Bone Angiogenesis

Angiogenesis plays a significant role in regulating bone development, since it is coupled with osteogenesis (bone formation) (Schipani et al., 2009; Kusumbe et al., 2014) and chondrogenesis (cartilage formation) (Gerber et al., 1999; Ramasamy et al., 2014; Romeo et al., 2019). Several pieces of evidence indicate a unique mode of blood vessel growth in bone compared to other tissues, which undergo sprouting or intussusceptive modes of angiogenesis. Sprouting angiogenesis is generally observed involving endothelial ‘tip cells’ at the leading front of new vessel sprouts, which exhibit long extensions or filopodia in the direction of angiogenic factors such as VEGF; ‘stalk cells’ follow and proliferate to elongate the sprout (Risau, 1997; Carmeliet et al., 2009; Ramasamy et al., 2016; Tiemeijer et al., 2018). This has been observed in cancer as well as the yolk sac and embryo, including during brain formation (Risau, 1997; Carmeliet et al., 2009). Intussusceptive angiogenesis is the splitting of existing vessels by columns of cells and is seen during the development of the lungs and heart (Skawina et al., 1994; Risau, 1997; Carmeliet, 2000).

**FIGURE 1:**
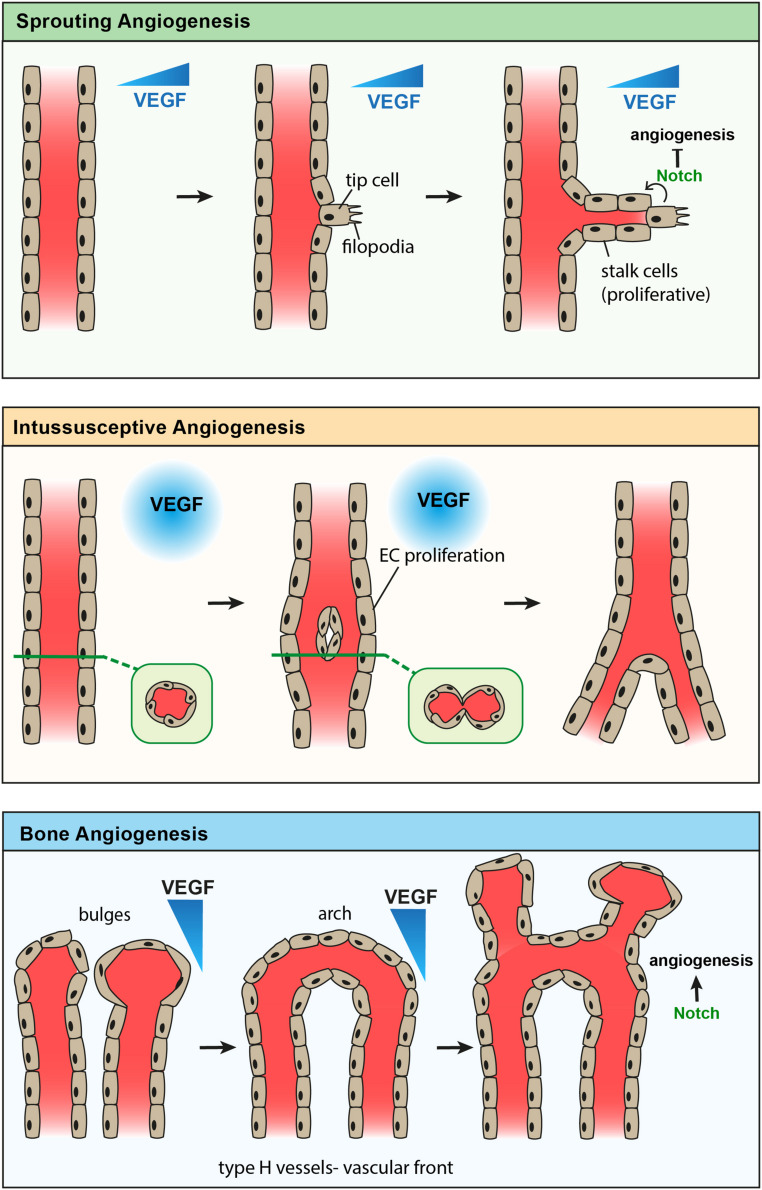
Comparison of known modes of angiogenesis. Sprouting angiogenesis **(top)** showing formation of an endothelial tip cell with filopodia in response to a gradient of VEGF. Stalk cells proliferate to elongate the new vessel branch, while Notch signaling from the tip cell inhibits further tip cell formation. Intussusceptive angiogenesis **(middle)** showing endothelial cell proliferation in response to high levels of VEGF, leading to the formation of a new endothelial vessel wall which splits one vessel into two. Angiogenesis of type-H vessels in bone **(bottom)** showing anastomosis of bulge structures, forming an arch structure. Endothelial cells proliferate to form new bulge structures in the direction of a VEGF gradient, while Notch signaling promotes angiogenesis in bone.

Scanning electron microscopy (SEM) of corrosion casts described both blind-ending sprouts and vessel splitting during the vascular invasion of the epiphyseal cartilage, suggesting both forms of angiogenesis are used in bone development (Skawina et al., 1994). Dye injection studies by Trueta and Morgan (1960) revealed the presence of columnarly arranged tubular structures with interconnecting loops in the metaphyseal region of long bones. Recent advanced high magnification imaging of metaphyseal type-H capillaries indicated unique bulge and columnar structures leading vessel growth in the angiogenic front. Bulge structures are prominent during developmental angiogenesis and mediate blood vessel growth in bones. Defective formation of bulge structures resulted in the inhibition of blood vessel growth (Ramasamy et al., 2014) similar to the suppression of tip cell formation (Gerhardt et al., 2003; Hellström et al., 2007). Compared to tip cells, bulge protrusions are multi-endothelial layered lumenised structures showing the active flow of blood. Lumenised bulges project filopodia into the surrounding chondrocyte matrix high in VEGF, reminiscent of the protrusions in tip cells during sprouting angiogenesis (Risau, 1997; Gerber et al., 1999; Ramasamy et al., 2016). Angiogenic blood vessel growth involves the emergence of bulges from distal arch/loop structures, which in turn anastomose to form new arches (Figure 1). Continuous formation of bulges and arches in the vascular front drive vessel growth and push blood vessels into the cartilage matrix. A subset of non-bone-resorbing osteoclasts termed VAOs was identified to mediate anastomoses of bulges during this blood vessel growth. Age-related decline in bulge formation is associated with the reduction of angiogenesis and manifest arch or loop structures in adults and aged bone metaphysis. Structures similar to intussusceptive angiogenesis were observed in columnar type-H capillaries; detailed investigations are still needed to understand the underlying mechanism. Irradiation injury leads to a significant increase in type-H vessels, including in the diaphysis, whilst the number of type-L vessels, which express low levels of Pecam1 and Emcn, decreases. Lineage tracing of type-H vessels shows that they give rise to type-L vessels (Kusumbe et al., 2014) during postnatal development. The expansion of type-L sinusoidal capillaries has not been well-studied to know whether they undergo independent angiogenesis or they are only matured endothelium.

The molecular mechanism mediating the growth of type-H capillaries differs from retinal vasculature. Notch signaling through Delta-like ligand 4 (Dll4) modulates the effects of VEGF in ECs, by further regulating angiogenesis (Ramasamy et al., 2016; Peng et al., 2020), while induction of EC Notch signaling in retinal ECs is antagonistic to sprouting and tip cell formation (Ridgway et al., 2006; Hellström et al., 2007; Lobov et al., 2007; Suchting et al., 2007; Ramasamy et al., 2016; Pitulescu et al., 2017; Tiemeijer et al., 2018). Tip cells express high levels of Dll4, inducing Notch signaling in neighboring ECs, causing them to downregulate VEGF receptors and become insensitive to the VEGF ligand (Tiemeijer et al., 2018). Similarly, bulge structures express high levels of Dll4, but EC-specific Notch gain of function leads to increases in arteriole and type-H vessel frequency (Hankenson et al., 2015; Kusumbe et al., 2016). Blood flow is a key regulator of bulge structures and type-H vessel phenotype, and it activates the Notch signaling pathway to control angiogenesis (Ramasamy et al., 2016). Notch plays a critical role in the maturation of type-H endothelium forming arteriolar vessels in the bone microenvironment (Kusumbe et al., 2016). Notch was found to be similarly important in remodeling and maturation of retinal vasculature (Ehling et al., 2013).

Blood vessels grow in response to tissue oxygen demand or hypoxic conditions. In hypoxia, bone osteoblasts and ECs accumulate HIFs, leading to transcriptionally increased expression of VEGF-A and other angiogenic genes (Brandi and Collin-Osdoby, 2006; Fong, 2008; Marenzana and Arnett, 2013; Peng et al., 2020). Higher levels of HIF-1α were detected in type-H endothelium compared to type-L, and further stabilization of HIFs in bone ECs increased the number of type-H capillaries in the bone (Kusumbe et al., 2014). Recently, the Hippo signaling pathway has been identified to regulate endothelial response to hypoxia in the bone (Sivaraj et al., 2020). Together, the unique nature of bone vasculature and its angiogenesis confers the need to understand the tissue-specific blood vessel growth for therapeutic targeting of blood vessels in diseases. In particular, the pivotal role of bone in regulating whole-body homeostasis indicates that systemic targeting of blood vessels could impact bone vasculature and raise complications associated with bone functions in the body.

## Organization of Blood Vessels in Bone

Like most organs, the mammalian bone vasculature consists of arteries from which oxygenated blood enters the bone and veins by which blood exits, connected through a network of capillaries (Marenzana and Arnett, 2013; Ramasamy, 2017; Gruneboom et al., 2019). Arteries in bone have been classified and discussed well in several studies (Rhinelander, 1974; Tomlinson and Silva, 2013; Prisby, 2017; Ramasamy, 2017; Chen et al., 2020). In general, arteries enter the bone and branch out into smaller arterioles to terminate in specialized type-H capillaries. Type-H capillaries are connected to type-L capillaries, which drain blood into smaller veins to flow out to a larger vein. Type-H capillaries, also called endosteal or transition zone capillaries (Acar et al., 2015) are localized in the metaphysis and endosteum regions, while type-L capillaries form dense, fenestrated, and highly branched sinusoidal network structures in the marrow cavity (Aharinejad et al., 1995; Kusumbe et al., 2014; Lafage-Proust et al., 2015; Ramasamy, 2017). Despite being structurally different, type-H and type-L vessels are interconnected and form a single vascular network in the central marrow cavity (Ramasamy et al., 2016). *Trans*-cortical vessels (TCVs) were recently identified in the cortical bone to provide short transport of blood flow in and out of the bone (Gruneboom et al., 2019).

The organizational structure of blood vessels has consequences for the patterns of both blood flow and oxygenation in the bone. Young adult long bones exhibit a largely centrifugal (inside to outside) blood flow pattern (Rhinelander, 1974; Tomlinson and Silva, 2013). The observation of centripetal (outside to inside) blood flow pattern in aged bones and fracture indicates the association of blood flow with bone health (Prisby, 2017). The mean partial pressure of oxygen (pO_2_) in the bone marrow of normal human volunteers is around 6.6%, comparable to other normal tissues where the median interstitial pO_2_ is 3–9% (Arnett, 2010; Marenzana and Arnett, 2013). Since arteries feed into the capillary network at the metaphysis and endosteum, those regions exhibit a higher pO_2_ than the central sinusoidal regions (Spencer et al., 2014; Lafage-Proust et al., 2015; Ramasamy et al., 2016). Mathematical models predict that pO_2_ may fall to as low as 1% in capillary-distant regions of the bone marrow (Marenzana and Arnett, 2013). Interestingly, this oxygenation profile is significantly altered in response to stresses such as irradiation and chemotherapy (Spencer et al., 2014). A major consumer of oxygen in the bone is hematopoietic cells; ablation of hematopoiesis significantly increases oxygen levels in the bone marrow space (Morrison and Scadden, 2014). Furthermore, pO_2_ in the cortex has also been shown to vary depending on the vicinity of either arteriolar or venular TCVs, with pO_2_ being higher in the former case and vice versa (Gruneboom et al., 2019). The pO_2_ heterogeneity in the bone may have significant functional consequences for different cell types and be an essential defining feature of multiple vascular niches in the bone (Arnett, 2010; Marenzana and Arnett, 2013; Ramasamy, 2017).

## Heterogeneity in Bone Endothelium

The organization of blood vessels and the oxygen profile in bones indicate the existence of cellular and functional differences in the bone vasculature. As discussed, EC markers Pecam1 and Emcn can be used to classify the bone capillary network into two morphologically distinct capillary subtypes with a unique distribution pattern in the bone marrow space. Columnar capillaries present in the metaphysis and endosteum, termed type-H, express high levels of both the markers, while type-L ECs in diaphyseal sinusoidal vessels display low levels of both markers (Kusumbe et al., 2014, 2016; Ramasamy et al., 2015; Peng et al., 2020). ECs forming type-H capillaries experience high blood velocity and higher pO_2_ levels compared to ECs lining type-L capillaries (Spencer et al., 2014; Kusumbe et al., 2016). Remarkably, the difference in EC junctions and permeability results in varying pO_2_ levels in their local microenvironments (Itkin et al., 2016).

Type-H capillaries are angiogenic blood vessels that form the leading front of blood vessel growth in the bone (Ramasamy et al., 2014, 2016), and express high levels of matrix metalloproteinases (MMPs) to regulate cartilage replacement during bone growth (Romeo et al., 2019). They are thus predominantly present in developing skeletal elements and decrease progressively in adults, with no apparent type-H blood vessel structures detected in aged bones. As a result, sinusoidal type-L capillaries localized in the diaphysis of developing bones occupy the entire bone marrow space in aged bones (Kusumbe et al., 2014). Age-related decline in type-H ECs is associated with bone loss and altered hematopoiesis. Reactivation of type-H vessels in aged mice led to new bone formation and hematopoiesis (Kusumbe et al., 2014, 2016; Ramasamy et al., 2016). However, the functions of type-L ECs have not been very well studied. Similarly, ECs forming TCVs, which provide a rapid exit route for neutrophils and likely other cells and factors from the bone, still need to be characterized (Gruneboom et al., 2019).

The heterogeneous bone vasculature contributes to the functional heterogeneity observed in the bone marrow microenvironment (Ramasamy, 2017). The differing properties of ECs result in supporting distinct perivascular mesenchymal cells and unique oxygen levels. For instance, arteriolar ECs specifically support PDGFRb+ perivascular cells and lower permeability of the vessels creates an environment low in oxygen and reactive oxygen species (ROS) (Itkin et al., 2016; Kusumbe et al., 2016). Thus, EC subtypes could define microenvironments within the bone compartment to regulate cellular and molecular composition, which in turn impacts functions of the bone in health and disease. A comprehensive characterization involving cellular, spatial, molecular, and functional roles of endothelial subtypes would contribute toward understanding the pleiotropic functions of the bone.

## Vascular Niches in Bone

### Hematopoietic Stem Cells

A niche is defined by a combination of anatomy and function, providing a unique local microenvironment which supports the maintenance or regulation of specific stem cell types (Morrison and Scadden, 2014). The bone marrow space has multiple niches, which are defined through physical factors such as pO_2_ and blood flow, as well as the types and expression profiles of endothelial and perivascular cells (Marenzana and Arnett, 2013). Bone marrow niches crucially support the entire lineage of hematopoietic and mesenchymal cells. The factors which define the hematopoietic stem cell (HSC) niche in bone have been studied extensively.

It was previously hypothesized that osteoblasts provide the HSC niche. However, imaging and osteoblast depletion experiments suggested that while osteoblasts are essential in forming bone ossicles where HSCs later reside, they do not directly maintain HSCs (Mizoguchi et al., 2014; Morrison and Scadden, 2014). Recently, the involvement of CD31+ CD105+ ECs in hematopoietic niche formation has also been suggested (Kenswil et al., 2018). *In vitro*, HSCs can be maintained by culturing with ECs; bone endothelium produces the best outcome, followed by endothelium from heart and liver (Ramasamy, 2017). Besides, irradiated mice transplanted with bone marrow EC cultures exhibit increased survival by supporting the regeneration of the HSC population (Sivan et al., 2019). The ability of ECs to maintain HSCs is diminished upon deletion of IL6R (the receptor for interleukin-6) and various endothelial secreted ‘angiocrine’ factors including CXCL12 and stem cell factor (SCF) (Ding et al., 2012; Sivan et al., 2019). The expression of these angiocrine factors by endothelia is Notch-dependent (Kusumbe et al., 2016; Ramasamy, 2017). Some of these factors, including SCF, are also provided by perivascular cells to sustain HSCs (Ding et al., 2012; Morrison and Scadden, 2014).

Multiple different vascular microenvironments have been identified to provide a supportive niche for HSC maintenance within the bone marrow space (Ramasamy, 2017). In long bones, catulin-α-GFP+ HSCs were found largely in the diaphysis, with approximately 85% within 10 μm of a sinusoidal vessel; in contrast, arterioles and transition (type-H) vessels were not associated with these HSCs (Acar et al., 2015). In addition, nearly all of these HSCs directly contacted a Leptin Receptor (LepR)+ CXCL12+ perivascular cell (Acar et al., 2015). Cxcl12-abundant reticular (CAR) cells colocalize with HSCs, and ablation of these perivascular cells depletes the HSC population (Morrison and Scadden, 2014).

In contrast, HSCs in the sternum preferentially associate near arterioles, while the association between HSCs and sinusoidal vessels was not significant (Kunisaki et al., 2013). Kusumbe and colleagues found a subset of type-H vessels with EphrinB2+ Sox17+ ECs which generate less permeable arteriolar vessels that support and are associated with HSCs in the endosteal region (Kusumbe et al., 2016). The former study also identified a subset of ‘bright’ nestin-GFP+ perivascular cells found exclusively along arterioles, which express higher levels of HSC niche-associated genes than the nestin-GFP+ cells associated with sinusoidal vessels (Kunisaki et al., 2013). Further pharmacological experiments suggested that arteriolar nestin-GFP+ cells promote HSC quiescence and safety from genotoxins (Kunisaki et al., 2013). Another study illustrated that nestin+ cells include both non-endothelial and endothelial lineage cells, both of which are potentially important for HSC maintenance (Ono et al., 2014). Identification of a subset of Hoxb5+ quiescent ‘long-term HSCs’ and their direct association with VE-cadherin+ arteriolar ECs (Chen et al., 2016) further confirmed the vascular microenvironment of HSCs.

Bone vasculature and HSC niches can also be affected by bone-external factors. In times of inflammatory stress, such as after administration of bacterial lipopolysaccharide, a visible increase in bone sinusoids and EC numbers were observed (Vandoorne et al., 2018). These highly permeable sinusoidal vessels support leukocyte migration and HSC proliferation (Itkin et al., 2016). This is accompanied by dilated blood vessel lumens and hypoxia immediately following the injection. Vascular leakiness and proliferation of hematopoietic stem and progenitor cells near permeable vessels are increased (Vandoorne et al., 2018). This highlights the ability of environmental signals and EC factors to regulate HSCs, as well as its physiological relevance. Sympathetic nerve fibers synapse on perivascular cells to regulate the HSC niche. It is thought that these may provide circadian regulation of Cxcl12 expression and HSC mobilization (Morrison and Scadden, 2014). Circulating cytokines, reproductive hormones, nutrition-related hormones, and thrombopoietin have also been identified to influence HSC niches (Morrison and Scadden, 2014). Thus blood vessels and their derived factors are key players in supporting HSCs in the bone marrow microenvironment.

### Mesenchymal Perivascular Cells

Blood vessels consist of an innermost layer of ECs, outside of which perivascular cells, also called mural cells or pericytes, are present. The types of perivascular cells in bone vary with blood vessel subtype. Type-L vessels have two main perivascular cell types: LepR+ stromal cells and CAR cells. Adipocytes expressing lipid droplet marker perilipin can also be detected adjacent to type-L capillaries. The perivascular cells of type-H vessels express PDGFRβ, NG2 and Nestin. Type-H capillaries are surrounded by osteoprogenitor cells which express markers such as Osterix (Osx) and Runx2. Perivascular cells surrounding arterioles express PDGFRβ, NG2 and Nestin similar to those found with type-H vessels but do not generate osteoprogenitor cells. Larger arteries have perivascular cells which express smooth muscle actin (α-SMA) (Ramasamy, 2017). However, it is still unclear the extent to which perivascular cell subtype markers overlap or are distinct populations.

The subtypes of cells and markers described above indicate mesenchymal lineage of perivascular cells. A perivascular origin for mesenchymal stem cells (MSCs) in various organs, including bone, has been described (Crisan et al., 2008). However, the presence of multiple mesenchymal subtypes in bones argues for the identification of multipotent perivascular cells. Many of the perivascular cells exhibit characteristics of MSCs *in vitro* and can differentiate into osteogenic, chondrogenic, or adipogenic lineages depending on the instructive signals (Doherty et al., 1998; Farrington-Rock et al., 2004). The mesenchymal cells surrounding the bone vasculature have been studied through lineage tracing. This shows that nestin+ cells found at arteries and type-H vessels represent early mesenchymal stem and progenitor cells (MSPCs), which can generate a wide range of cells types in the bone marrow stroma and bone lineages (Mizoguchi et al., 2014). LepR+ cells around type-L vessels preferentially contribute to the bone lineage during early development, but the adipocyte lineage in adults (Zhou et al., 2014). Similarly, Osx+ cells are associated with a wide range of lineages during neonatal development, including bone marrow stroma, bone, chondrocyte, and adipocyte lineages (Mizoguchi et al., 2014; Ramasamy, 2017). However, they are considered to be osteoblast precursors in adults, which play an essential role in active bone repair and remodeling (Mizoguchi et al., 2014; Peng et al., 2020). Meanwhile, CAR cells have been associated with supporting HSCs and the HSC niche (Ding et al., 2012; Mizoguchi et al., 2014; Morrison and Scadden, 2014). Perivascular mesenchymal subtypes and their vascular niches in bones have been summarized in Table 1.

**TABLE 1:** Characteristics and functions of perivascular cell types in bone marrow.

Cell type	Blood vessel niche	Markers	Functions	References
LepR+ Stromal Cells	Type-L	LepR, Angpt1, PDGFRα	Give rise to bone cells and adipocytes; proliferate after injury to regenerate bone	Sacchetti et al., 2007; Ono et al., 2014; Zhou et al., 2014, 2015; Ramasamy, 2017
Cxcl12-Abundant Reticular (CAR) Cells	Type-L	Cxcl12, SCF, PDGFRβ, Foxc1, LepR, PDGFRα	Supportive niche for HSCs; give rise to adipocyte and osteoblast lineages	Sugiyama and Nagasawa, 2012; Ding and Morrison, 2013; Omatsu et al., 2014
Adipocytes	Type-L	Perilipin, PPARγ, adiponectin, FABP4, LPL	Endocrine secretion; lipid metabolism; HSC regulation	Cawthorn et al., 2014; Bukowska et al., 2018
Osteoprogenitors	Type-H	Osterix, Runx2	Give rise to osteoblasts in adults	Kusumbe et al., 2014; Ramasamy, 2017
Periarteriolar/Nestin+ MSCs	Type-H, Arteriole	Nestin-GFP, PDGFRβ, NG2, PDGFRα, CD51, CD146, CXCL12, SCF	Give rise to multiple mesenchymal lineages, including osteoprogenitors; supportive niche for HSCs; promote HSC quiescence	Méndez-Ferrer et al., 2010; Kunisaki et al., 2013; Pinho et al., 2013; Itkin et al., 2016; Kusumbe et al., 2016; Ramasamy, 2017
Smooth Muscle Cells	Arteriole, Artery	α-SMA	Regulate arterial vasomotor functions	Lund et al., 2013; Kusumbe et al., 2014; Ramasamy, 2017

The importance of blood vessel heterogeneity in regulating mesenchymal cell fate decisions has not yet been well understood. However, blood vessels transport nutrients to regulate tissue metabolism and fate of mesenchymal cells. A recent study from Carmeliet lab showed limited growth of blood vessels in fracture sites promotes chondrogenesis compared to osteogenesis (van Gastel et al., 2020). Absence of a functional vascular bed to supply oxygen keeps cartilage tissue avascularised and maintains chondrocytes in profound hypoxia (Schipani et al., 2001). Hypoxia and HIF signaling are critical players of collagen synthesis in chondrocytes (Stegen et al., 2019). Capillary subtypes in bone were identified to show different permeability to allow varying oxygen levels in their local microenvironments (Itkin et al., 2016). Measurement of local oxygen concentration confirmed the presence of heterogeneity in oxygen levels near vasculature (Spencer et al., 2014). These studies indicated the coexistence of endothelial heterogeneity and diverse oxygen levels in the bone marrow compartment. Further, these findings indicate the importance of blood vessels in regulating the tissue metabolism and cellular composition of local microenvironments.

## Endothelial Role in Bone Repair

Bone exhibits high regenerative potential, and many of the factors which couple angiogenesis and osteogenesis in development and remodeling are also involved in bone repair (Marenzana and Arnett, 2013; Kusumbe et al., 2014; Hankenson et al., 2015; Sivan et al., 2019). When injured, the bone is encapsulated by a hematoma in the fracture area, with local hypoxia in the region (Blevins, 1968; Hankenson et al., 2015; Sivan et al., 2019). In rabbits, the observed pO_2_ post-fracture is 1–3% (Marenzana and Arnett, 2013). This hypoxia causes ECs to upregulate BMP-2 to promote osteogenesis (Hankenson et al., 2015; Sivan et al., 2019). Besides, osteoblasts accumulate HIF-1α, leading to VEGF-A production, which further enhances angiocrine BMP production; HIF-1α in ECs is also shown to be important to the healing process (Brandi and Collin-Osdoby, 2006; Wang et al., 2007; Bahney et al., 2015). Injury-mediated angiogenesis has also been suggested to guide osteoblast precursors to the injury site (Maes et al., 2010; Hankenson et al., 2015).

Both endochondral and intramembranous ossification processes are involved in fracture healing (Tomlinson and Silva, 2013). Most non-stabilized fractures heal through endochondral ossification (Behonick et al., 2007; Hu et al., 2017). However, stress fractures, as well as stabilized fractures and cortical defects, are more likely to heal through intramembranous ossification (Behonick et al., 2007; Tomlinson and Silva, 2013). Both the initial inflammatory and following anti-inflammatory stages of the hematoma promote the production of angiogenic factors including VEGF, angiopoietin-1, PDGF, TGFβ, and epidermal growth factor (EGF) from leukocytes (Bahney et al., 2015). Other factors which promote both MSC proliferation or differentiation and angiogenesis in healing include SDF1 (and its receptor CXCR4) and basic FGF (bFGF) (Hankenson et al., 2015; Zhang et al., 2017). MSCs in the early callus differentiate to chondrocytes which further promote vascularization and bone formation through the secretion of BMP, MMP-13, alkaline phosphatase, VEGF, and placental growth factor (PIGF) (Bahney et al., 2015). Chondrocytes in the callus are also found to release anti-angiogenic factors which limit blood vessel growth. Interestingly, mice lacking functional MMP-9 have a distinct fracture repair phenotype – an excessively large callus forms, and healing occurs through the endochondral mechanism rather than by the intramembranous pathway (Ortega et al., 2004). It is also suggested that the production of MMPs by type-H vessels for cartilage resorption may also be an essential part of fracture repair (Romeo et al., 2019). Overall, the vasculature closely interacts with the injured tissue in supporting the repair mechanism (Blevins, 1968; Sivan et al., 2019).

Blood flow has historically been linked to bone healing. Orthopedic surgeons and researchers observed that increased blood flow during tibial fracture healing aids bone mineralization, and that vessel walls appear to have osteogenic properties (Rhinelander, 1974; Trueta, 1974). Methods of artificially increasing blood flow during early stages of fracture are used in treatment, whilst bone allografts have a failure rate of 16–35% partly attributed to the low vascular invasion of grafted tissue (Blevins, 1968; Bahney et al., 2015). Furthermore, it has been observed that blood flow in bone increases up to sixfold following fracture due to vasodilatation (Tomlinson and Silva, 2013). However, the relationship between angiogenesis and bone repair may be more nuanced. The anti-angiogenic agent endostatin impedes hard callus formation but promotes soft callus formation (Brandi and Collin-Osdoby, 2006; Tomlinson and Silva, 2013). Besides, vasoconstrictors such as nicotine inhibit bone regeneration but promote angiogenesis, although other studies find that smoking delays fracture healing by inhibiting angiogenesis either directly or indirectly (Marenzana and Arnett, 2013; Bahney et al., 2015). This suggests a more complex balance or interplay between pro- and anti-angiogenic factors in the healing process.

## Vascular Functions in Bone Physiological Remodeling

Bone is a highly dynamic tissue which undergoes constant remodeling throughout life (Eriksen, 2010; Davis et al., 2015). Remodeling is required not only to adapt to the changing physiological demands on the skeleton throughout life but also to repair microdamage which occurs due to the high level of mechanical stress bones experience (Land and Schoenau, 2008; Eriksen, 2010). Bone remodeling is a balance between bone resorption and bone formation, and both of these processes depend on and are closely coupled to the bone vasculature (Eriksen, 2010; Marenzana and Arnett, 2013; Davis et al., 2015).

### Age

Reduction of type-H vessels and arteries in aging long bones is associated with the change in blood flow to centripetal (outside to inside) pattern from centrifugal (inside to outside) flow in young bones (Rhinelander, 1974; Tomlinson and Silva, 2013; Prisby, 2017). Centripetal flow patterns can also be observed in some injury situations such as destruction of the principle nutrient artery (PNA) or bone fracture. In this case, blood flow from the periosteum can increase to compensate for the loss of central blood flow (Blevins, 1968; Tomlinson and Silva, 2013). Similarly, aging causes the hemodynamic pressure of the central diaphysis to fall below that of the arterial periosteum (Bridgeman and Brookes, 1996; Guderian et al., 2019). It is also observed that the total speed and volume of blood in the bone decrease with age, causing marrow pH and pO_2_ to fall (Bridgeman and Brookes, 1996). The ratio of blood flow and pooling in the femoral head versus its diaphysis has been identified to fall with age (Hamaguchi et al., 2006). These changes may impact hematopoiesis, impair bone regenerative ability, as well as increase the risk of osteoporosis in older people (Bridgeman and Brookes, 1996; Hamaguchi et al., 2006; Marenzana and Arnett, 2013). Age-related changes in blood vessels and vascular niches impact hematopoietic stem cells (HSCs) (Morrison and Scadden, 2014; Kusumbe et al., 2016; Pinho and Frenette, 2019). Involvement of vascular niches has been identified to contribute to age-associated myeloid bias in hematopoiesis (Ho et al., 2019). Moreover, several reports indicate the role of vascular niches in blood cancers (Passaro et al., 2017; Duarte et al., 2018) and metastasis (Kusumbe, 2016; Singh et al., 2019) which are discussed in other associated reviews in this special edition.

### Gender

Studies on biological differences between male and female bones indicate estrogen as a major player in regulating gender-specific changes. Part of the bone phenotype after menopause is undoubtedly a direct effect of reduced estrogen levels on the bone (Rizzoli and Bonjour, 1997). Blood flow to the femoral head is more significant in females than in males before the age of fifty, but this decreases with age to a much greater extent in females (Hamaguchi et al., 2006), strikingly correlating with the average age of menopause, which is 48.8 years globally (Davis et al., 2015). Estrogen function in osteoblasts and osteoclasts has been widely studied. Estrogen receptors are present on ECs (Brandi et al., 1993), and estrogen is linked to enhanced angiogenesis during pregnancy (Osol et al., 2019). Signaling through estrogen receptor alpha (ERa) is thought to increase the bioavailability of EC-derived nitric oxide (NO) (Osol et al., 2019). NO promotes vasodilatation, and its deficiency causes endothelial dysfunction (Jin and Loscalzo, 2010). The inhibition of NO or NO synthase in rats has a similar bone loss and vasoconstriction phenotypes as ovary removal (Wimalawansa et al., 1996). Furthermore, NO concentrations are also known to affect both osteoclast and osteoblast growth and activity (Eriksen, 2010). This suggests that postmenopausal decline in estrogen may impact bone blood flow and hence bone remodeling through the endothelial NO pathway.

Interestingly, many of the same risk factors associated with endothelial dysfunction and cardiovascular disease, including age, hyperparathyroidism, and hypertension, are also correlated with bone loss and osteoporosis. This further supports a link between vasculature and bone maintenance. However, it is important to note that despite common risk factors, males are more likely to develop cardiovascular disease, and females osteoporosis (Alagiakrishnan et al., 2003). This is because estrogen is protective against cardiovascular disease pre-menopause, while males have a higher baseline bone mass (Mendelsohn and Karas, 1999). Although sex hormone deficiency in both males and females is known to lead to bone loss, the bone phenotypes caused by sex hormone changes are not well-studied outside the context of menopause. Patients undergoing estrogen and anti-androgen treatment exhibit lower bone thickness and turnover, but show no bone loss (Lips et al., 1989). Testosterone treatment conversely led to an increase in cortical bone thickness, but lower bone mineral density (BMD) (Van Caenegem et al., 2012). A meta-analysis found no effect on BMD after testosterone treatment but did report an increase after estrogen treatment (Fighera et al., 2019). Unfortunately, studies into bone vasculature changes after HRT are lacking, although we can speculate that estrogen treatment may lead to increased bone blood flow, thus possibly supporting the observed increase in BMD.

Levels of estrogen are high during pregnancy, produced first by the ovaries and later the placenta; the placenta also releases VEGF (Kumar and Magon, 2012; Osol et al., 2019). These lead to global vascular changes, including increased NO, reduced vascular tone, and angiogenesis (Osol et al., 2019). Meanwhile, pregnancy is found to be a high bone turnover state, with an overall loss of trabecular bone in the spine and pelvis, but increased BMD in the limbs (Naylor et al., 2000). Mothers also lose 3–7% of their BMD during breastfeeding, although recovery after weaning is usually swift and complete (Kalkwarf and Specker, 2002). Changes in the bone vasculature during pregnancy are not well-studied; however, the role of blood vessels in the skeletal changes during pregnancy is predicted. High levels of estrogen and VEGF promote bone formation and increased BMD, whilst the increased mechanical load from carrying the developing fetus may further promote bone formation.

### Hormones

Apart from estrogen, many other hormones present in the body affect bone remodeling and physiology. Parathyroid hormone (PTH) plays a significant role in regulating calcium homeostasis through bone resorption and calcium retention (Prisby, 2017). Hyperparathyroidism, a state of elevated systemic PTH, is associated with osteoporosis and increased bone turnover (Eriksen, 2010; Davis et al., 2015). However, intermittent PTH administration conversely supports bone formation (Hock and Gera, 1992). This is thought to be due to its independent vasodilatory effects through EC NO signaling, thus increasing bone blood flow, as well as possibly enhancing angiogenesis specifically into areas of active bone remodeling (Prisby et al., 2011; Romeo et al., 2019).

Pharmacological treatments of glucocorticoids (GCs) to reduce inflammation and immune response result in osteoporosis (Weinstein, 2001). Endogenous GCs were found to reduce angiogenesis, vascularity and blood flow in bones (Weinstein, 2010). GC treated mice showed reduced type-H capillaries (Lane et al., 2018) and mediated the generation of osteonecrosis in mice (Weinstein et al., 2017). The increase in levels of endogenous GCs with age indicates its role in the decline of bone mass (Van Cauter et al., 1996; Weinstein, 2010). However, the role of GCs on bone vasculature and its importance in bone formation needs further understanding to use GC-based treatment regimens efficiently.

Osteocalcin, a peptide hormone released by osteoblasts during bone formation, was identified to regulate blood vessel growth in angiogenesis animal models (Cantatore et al., 2005). Importance of osteocalcin indicates its potential role in bone formation during development and repair. Vitamin D is involved in calcium regulation and is essential for skeletal maintenance (Davis et al., 2015). However, vitamin D receptors are detected in ECs, knockout of which reduces NO bioavailability and thus impairs vasodilatation (Andrukhova et al., 2014). Calcitonin, produced in the thyroid gland, is also known to have anti-resorption activity through its direct effect on osteoclasts and is used to treat osteoporosis (Naot and Cornish, 2008). Calcitonin’s role in controlling vasomotor functions of blood vessels in bones has been described (Prisby, 2017). Calcitonin gene-related peptide (CGrP), a protein produced through alternative splicing of the calcitonin gene, induces vasodilatation (Lundgaard et al., 1997). Finally, oxytocin directly stimulates bone remodeling through activating osteoblast and osteoclast differentiation (Tamma et al., 2009). Besides, it also supports NO synthesis and is known to have a vasodilatory effect on small arteries (Tamma et al., 2009; Rabow et al., 2018). Therefore, vasomotor effects could be one mechanism by which these three hormones support bone formation.

**FIGURE 2:**
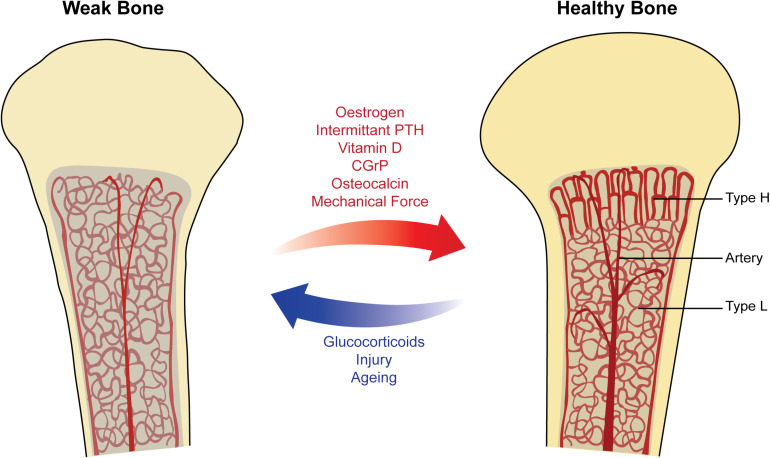
Factors affecting vasculature in physiological remodeling of bone. Healthy bone **(right)** showing columnar type-H vessels in the metaphysis and sinusoidal type-L vessels in the diaphysis. The bone marrow has a strong blood supply and is well-oxygenated, supporting the process of bone remodeling and increasing bone mineral density (BMD). Weak bone **(left)** showing sinusoidal type-L vessels filling the marrow space, with decreased blood flow and oxygenation. These factors lead to a reduction in hematopoiesis and increased risk of osteoporosis. Arrows (middle) show selected physiological factors known to promote the strong healthy bone state (red) and the weak bone state (blue).

Changes in non-sex hormones may play a notable role in the skeletal changes observed throughout age and pregnancy. For instance, falling vitamin D synthesis and secondary hyperparathyroidism are associated with old age, thus being another factor in reduced bone formation in elderly people (Davis et al., 2015). Meanwhile, pregnancy is associated with low PTH and vitamin D levels, but high levels of calcitonin; oxytocin is also high during parturition and breastfeeding (Land and Schoenau, 2008; Kumar and Magon, 2012). These hormonal changes will also affect the balance of bone resorption and formation throughout pregnancy to some degree through their vascular effects. Finally, other systemic hormones with vascular effects may also indirectly affect bone remodeling (Figure 2). For instance, rat bone arterioles are sensitive to noradrenaline, a vasoconstrictor stress hormone (Fleming et al., 2001). Additionally, exercise can stimulate adrenaline production, which is also known to have systemic vasomotor effects, thus being yet another possible link between muscle use and bone maintenance (Prisby, 2017).

### Physical Activity

The link between skeletal muscle use and bone blood flow also demonstrates that bone remodeling is responsive to the physical demands of an individual (Alagiakrishnan et al., 2003; Davis et al., 2015). Preterm infants tend to have lower bone mass and density at their expected term than term babies, a difference that was suggested to be partially due to the reduced mechanical stimulation after birth versus *in utero* (Land and Schoenau, 2008). Similarly, newborns with intrauterine-onset congenital neuromuscular diseases, which significantly decreases the mechanical force experienced by bones, show a delay in periosteal bone deposition and reduced cortical bone thickness at birth (Rodríguez et al., 1988). Several studies on sports personnel indicate the direct correlation between physical activity and bone health (Forwood and Burr, 1993; Kohrt et al., 2004) which confirm the findings on muscle use and blood flow. Similarly, exercise and weight-bearing training were shown to reduce bone loss and improve bone mass in adults and postmenopausal women (Dalsky et al., 1988; Guadalupe-Grau et al., 2009). Physical activity improves bone capillarity in exercise-trained aged rats compared to control animals (Viboolvorakul et al., 2009) which could occur through the promotion of blood flow and type-H capillaries in bones (Ramasamy et al., 2016). Apart from blood flow, the role of mechanical forces on bone vasculature and angiogenesis needs further understanding.

## Conclusion

Dynamic remodeling of the skeletal system in mammals permits interaction of bones with the whole body to maintain homeostasis, and to respond to physiological changes and diseases. For instance, an increase in osteoclast activity helps in maintaining calcium levels by moving calcium from the bone when there is a demand (Rowe et al., 2020). Likewise, multiple cell types present within the bone contribute toward the functional interaction of the skeletal system with other organs. Importance of bone-derived factors such as osteocalcin and Lipocalin-2 in the regulation of systemic functions has been an intensive field of research (Mera et al., 2018; Chen et al., 2020). Changes in the contribution of a particular cell type in the bone microenvironment would result in specific functional alterations of bone. Distribution of cells in their local microenvironment and interaction with neighboring cells are essential for the survival and efficient functioning of cells. Recognition of EC functions in maintaining different cell types and their microenvironments have significantly motivated the interest toward understanding blood vessel heterogeneity and microenvironments that regulate bone physiological functions. However, the mechanisms and reasons for the generation and regulation of various microenvironments are still in the early stages of understanding. The involvement of intrinsic endothelial factors in generating vascular heterogeneity cannot be excluded completely. Besides, studies on systemic conditions that alter skeletal microenvironments would generate relevant knowledge on factors and their cell-specific functions. EC metabolism has recently gained significant interest in regards to organ-specific and tissue-specific functional specialization of blood vessels. The central role played by blood vessels in the bone physiology indicates the importance and urgent need to study vascular heterogeneity to understand the existence of multiple microenvironments within the bone.

## Author Contributions

MH prepared the first draft of the review and the figure. SR conceptualized and wrote the review. Both authors contributed to the article and approved the submitted version.

## Conflict of Interest

The authors declare that the research was conducted in the absence of any commercial or financial relationships that could be construed as a potential conflict of interest.
